# Daily Heart Rate Variability before and after Concussion in an American College Football Player

**DOI:** 10.3390/sports7050097

**Published:** 2019-04-27

**Authors:** Andrew A. Flatt, Gary B. Wilkerson, Jeff R. Allen, Clay M. Keith, Michael R. Esco

**Affiliations:** 1 Georgia Southern University, Department of Health Sciences and Kinesiology, Biodynamics and Human Performance Center, 11935 Abercorn, St. Savannah, GA 31419, USA; 2 Department of Kinesiology, Exercise Physiology Laboratory, University of Alabama, Tuscaloosa, AL 35487, USA; mresco@ua.edu; 3 Graduate Athletic Training Program, University of Tennessee at Chattanooga, Chattanooga, TN 37403, USA; gary-wilkerson@utc.edu; 4 Department of Athletics, Sports Medicine, University of Alabama, Tuscaloosa, AL 35487, USA; jallen@ia.ua.edu (J.R.A.); ckeith@ia.ua.edu (C.M.K.)

**Keywords:** autonomic, cardiac-parasympathetic, traumatic brain injury, sports medicine, sports science

## Abstract

This case report demonstrates the effects of sport-related concussion (SRC) on heart rate variability (HRV) in an American college football player. Daily measures of resting, ultra-short natural logarithm of the root mean square of successive differences (LnRMSSD), subjective wellbeing, and Player Load were obtained each training day throughout a 4-week spring camp and 4 weeks of preseason training. SRC occurred within the first 2 weeks of the preseason. During spring camp and preseason pre-SRC, the athlete demonstrated minimal day-to-day fluctuations in LnRMSSD, which increased post-SRC (LnRMSSD coefficient of variation pre-SRC ≤ 3.1%, post-SRC = 5.8%). *Moderate* decrements in daily-averaged LnRMSSD were observed post-SRC relative to pre-SRC (Effect Size ± 90% Confidence Interval = −1.12 ± 0.80), and the 7-day rolling average fell below the smallest worthwhile change for the remainder of the preseason. LnRMSSD responses to SRC appeared similar to trends associated with stress and training fatigue. Therefore, performance and sports medicine staff should maintain regular communication regarding player injury and fatigue status so that HRV can be interpreted in the appropriate context. Detection and monitoring of autonomic dysregulation post-SRC may require near-daily assessment, as LnRMSSD showed greater daily fluctuations rather than chronic suppression following the head injury.

## 1. Introduction

Player monitoring strategies and data analysis techniques in collegiate football are generally managed by performance staff. Microsensor-derived training load, subjective well-being, and ultra-short heart rate variability (HRV) represent a few of the measures currently being used by American college football teams in an effort to optimize player health and performance [1,2,3,4]. However, effective use of player monitoring tools such as HRV requires interpretation of the data in appropriate context given its sensitivity to a variety of physiological, psychological, and environmental factors [5].

High speed collisions put players at risk for sustaining sport-related concussion (SRC), which accounts for 8% of all reported American football injuries [6]. Emerging evidence from advanced neuroimaging and neurophysiological studies strongly suggest that SRC can have long-lasting adverse effects on brain function [7,8]. Interrelationships among neurometabolic, hormonal, and mechanical factors present an exceedingly complex challenge to clinicians who must make decisions in the management of highly heterogeneous manifestations of SRC [9]. Symptoms are poor indicators of neurological recovery, and standard clinical tests have been found inadequate for detection of subtle abnormalities that can persist beyond return to sport participation [10,11]. A number of published reports have established HRV as a reliable marker of autonomic dysregulation after SRC [10,12,13], potentially confounding interpretation of fatigue-induced alterations among collision-sport athletes [1,2,14,15,16]. Daily HRV averaged over a period of time (e.g., 1–2 weeks) reflects mean parasympathetic activity while its coefficient of variation (CV) reflects the magnitude of daily HRV fluctuations. Reductions in mean HRV and increases in its CV are commonly observed in fatigued team-sport and anaerobic-event athletes [15,17,18], reflecting withdrawn and unstable cardiac-parasympathetic activity. Thus, alterations in HRV patterns induced by training fatigue or SRC may be indistinguishable in the absence of added contextual information.

Inter-individual differences and lability in HRV make the availability of pre-injury baseline data essential for identification of persisting SRC effects, which has been presented in only one recent report [19]. Evidence of an association between SRC and altered HRV has been derived from time-consuming measurement protocols and sophisticated instrumentation, limiting its wide-spread implementation among sports teams. Further investigation into the usefulness of more convenient and time-efficient procedures for obtaining HRV that are currently being used for monitoring training adaptations in collision-sport athletes is needed [1,2,14,15,16]. Moreover, given that members of the performance staff (e.g., strength and conditioning coaches) are often managing the HRV data of players, a greater understanding of the effects of SRC on HRV is critical to recognize responses that may require attention from the sports medicine staff. Thus, the purpose of this case presentation is to report longitudinal changes in HRV from measurements acquired before and after the occurrence of SRC in a college football player.

## 2. Materials and Methods

### 2.1. Participant

This observational case report reviews data acquired from a Division-1 college football player (non-lineman) in the weeks before and after a SRC. Informed consent and ethical approval (6253) was obtained to use the acquired data for research purposes.

### 2.2. Procedures

#### 2.2.1. Observation Period

The player was monitored throughout spring camp (mid-March–mid-April) and preseason football training (August–September). Resting HRV, subjective indicators of recovery status, and Player Load were obtained each training day throughout the observation periods. Variables were compared across three time-points as follows: (1) spring camp, (2) pre-season, pre-SRC (Pre-SRC) and (3) pre-season, post-SRC (Post-SRC). SRC-indicators were collected following SRC-occurrence and were compared to pre-existing baseline values. 

#### 2.2.2. Heart Rate Variability

Resting-HRV was obtained at least 60-min post-prandial and before each football training session throughout spring camp and preseason football training, as previously described [1]. Briefly, HRV was measured for 1 min following a 1-min stabilization period in the seated position. HRV was derived from finger-pulse plethysmography and accompanying application (ithlete^TM^, HRV Fit Ltd., Southampton, UK) on a tablet device, previously shown to provide acceptable agreement with electrocardiograph-derived HRV [20]. One-min HRV recordings obtained at the training facility in collision-sport athletes under similar conditions have demonstrated acceptable relative (Intraclass Correlation= 0.90) and absolute inter-day reliability (CV = 7.65%) [16]. After completion of the recording, the application automatically computes and displays the vagal-HRV parameter, the natural logarithm of the root mean square of successive differences (LnRMSSD), which is multiplied by twenty to fit an approximate 100-point scale. The application uses a previously described processing algorithm to filter artefacts and ectopic beats [1]. 

#### 2.2.3. Training Load

External training load was quantified via 100 Hz tri-axial accelerometer (Catapult Innovations, Melbourne, Australia), fastened to the shoulder pads between the scapulae as previously described [1]. Total Player Load was selected for analysis because it reflects total external workload during training and is expressed as the square root of the sum of the squared instantaneous rate of change in acceleration in each vector and divided by 100 [21].

#### 2.2.4. Subjective Indicators of Wellbeing

A daily wellness questionnaire adapted from McLean et al. [22] was completed following each HRV recording. The player rated his perceived level of sleep quality, fatigue, muscle soreness, stress, and mood on a 1–9 scale [23]. A rating of 5 represented feeling “okay” while greater and lower numbers reflected improvements or decrements, respectively, in a given parameter [23]. 

#### 2.2.5. Concussion Testing

The cognitive assessment (Standardized Assessment of Concussion) and symptom scale of the second version of the Sport Concussion Assessment Tool (SCAT2) were used to document baseline status approximately 18 months prior to concussion occurrence. The corresponding components of the third version (SCAT3), which were unchanged in format from the previous versions, were used to document changes in symptom number, symptom severity, and cognitive function after concussion occurrence.

### 2.3. Statistical Analysis

**Figure 1:**
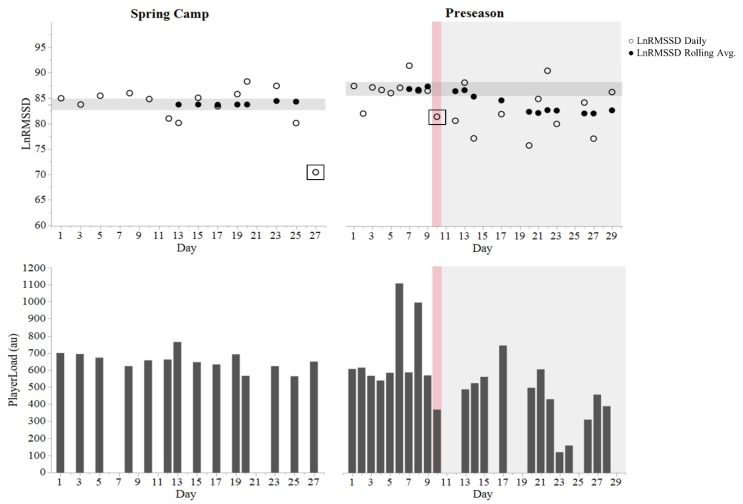
Natural logarithm of the root mean square of successive differences (LnRMSSD) and Player Load values throughout spring camp and preseason training. The horizontal shaded zone represents smallest worthwhile change thresholds for LnRMSSD. Red shade represents day of concussion. Grey shade represents the post-concussion period. Boxed data points represent scrimmage days.

LnRMSSD was analyzed by determining if daily values or the ~7-day rolling average positively or negatively exceeded the smallest worthwhile change (SWC). The SWC thresholds were calculated as ± 0.5 of the coefficient of variation from the first seven measures obtained from spring camp and again from the first seven measures of preseason camp [24,25]. The mean (LnRMSSD_M_) and coefficient of variation (LnRMSSD_CV_) from spring camp (13 recordings), Pre-SRC (9 recordings), and Post-SRC (11 recordings) were calculated. LnRMSSD values from scrimmage days were excluded due to potential effects of pre-competitive anxiety on autonomic activity (boxed data points in Figure 1). Pre-competition arousal can reduce HRV and therefore may obscure the typical day-to-day variation from training that we aimed to capture for comparison to post-SRC responses. Cohen’s *d* effect sizes (ES) [26] ± 90% confidence interval (CI) were used to compare LnRMSSD_M_ and Player Load values between time-points using the following qualitative thresholds: <0.2 = *trivial*; 0.2–0.59 = *small*; 0.6–1.19 = *moderate*; 1.2–1.99 = *large*; and >2.0, *very large* [24]. All analyses were carried out using JMP Pro 12 (SAS Institute Inc., Cary, NC, USA) and Excel 2016 (Microsoft Office, Redmond, WA, USA).

## 3. Results

LnRMSSD and Player Load values from spring camp and preseason can be viewed in Figure 1. LnRMSSD_M_ from spring camp, Pre-SRC, and Post-SRC were 84.3 ± 2.6, 86.8 ± 2.4, and 82.4 ± 4.8, respectively. Reductions in LnRMSSD_M_ at Post-SRC were *moderate* (ES = −1.12 ± 0.80) relative to pre-SRC and *small* (ES = −0.50 ± 69) relative to spring camp. LnRMSSD_CV_ from spring camp, Pre-SRC, and Post-SRC was 3.1%, 2.7%, and 5.8%, respectively. 

Player Load values from spring camp, Pre-SRC, and Post-SRC were 655 ± 54, 542 ± 66, and 441 ± 169, respectively. Player Load from post-SRC was *moderately* lower than pre-SRC (ES = −0.76 ± 0.68) and *largely* lower than spring camp (ES = −1.73 ± 0.74).

**Figure 2:**
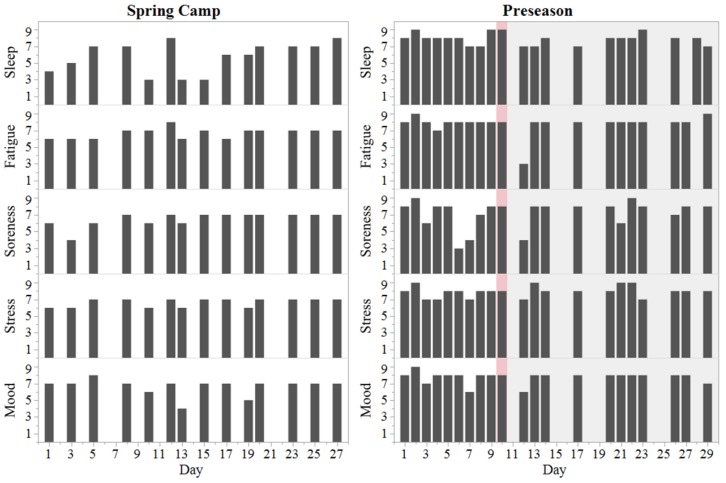
Subjective indicators of well-being throughout spring camp and preseason training. Red shade represents day of concussion (Day 10). Grey shade represents the post-concussion period.

Decrements in some markers of well-being were reported within 48 h post-SRC but returned to pre-SRC ratings thereafter (Figure 2). Restricted (i.e., non-contact) football participation was performed as tolerated and without exacerbation of symptoms by 72 h post-SRC. Unrestricted football participation occurred at 7 days post-SRC. The athlete denied ever having sustained a concussion prior to the occurrence documented in this report. Concussion status indicators can be viewed in Table 1.

## 4. Discussion

This case report documents alterations in serial measurements of HRV acquired from a college football player following SRC. A novel aspect of this report is the longitudinal observation period, enabling the establishment of baseline LnRMSSD trends to serve as a reference point for post-SRC data comparison. The main finding was that meaningful reductions in LnRMSSD persisted beyond return-to-play clearance and were graphically similar to trends exhibited by overreached athletes.

The athlete presented a remarkably low LnRMSSDcv during spring camp (3.1%) and during the pre-SRC period (2.7%). Average LnRMSSDcv values among players of the same position group during spring camp were 8.5 ± 1.7% [1]. Low LnRMSSDcv generally reflects high fitness and positive adaptation to training among collision-sport athletes [15,17]. Conversely, an increased LnRMSSDcv coupled with a reduced LnRMSSD_M_ (demonstrated by the athlete Post-SRC) have been associated with high perceived fatigue and poor training adaptation [17,18,23]. Whether Post-SRC LnRMSSD changes were SRC- or training-induced is inconclusive due to the observational nature of this report. Peak Player Load values occurred on days 6 and 8 of the preseason (Pre-SRC) and represent two-a-day practice sessions (Figure 1). Cardiac-parasympathetic recovery from intensive training takes place within 24–48 h post-exercise [27]. In addition, highly-fit athletes have been shown to maintain stable LnRMSSD and subjective self-report measures of recovery despite significant (*p* < 0.05) increments in training load during preparatory training [15]. Therefore, reductions in LnRMSSD subsequent to day 10 (Post-SRC) were unlikely associated with the acute spikes in Player Load from days 6 and 8. In support of this postulation, Post-SRC Player Load values were *moderately* reduced relative to Pre-SRC, and subjective indicators were not dramatically altered (Figure 2), which generally facilitates the return of LnRMSSD to or above baseline [18]. Thus, we contend that the changes in LnRMSSD were likely SRC-induced rather than training fatigue-related. 

Daily LnRMSSD was not chronically suppressed Post-SRC, but exhibited marked fluctuations (Figure 1). Consequently, the Pre-SRC vs. Post-SRC 90% CI of the ES ranged from *small*–*large*. Reliance on single-time point HRV measures Post-SRC may therefore be insufficient and misleading to practitioners for assessing the effects of SRC on autonomic function. For example, if LnRMSSD was acquired in isolation on day 13, 21, 22, 26, or 29, one might falsely conclude that SRC was no longer or only minimally affecting cardiac-autonomic activity. Whereas if LnRMSSD was obtained from any other day Post-SRC, converse interpretations would be made. Relative to isolated recordings, serial measures improved the diagnostic ability of LnRMSSD for identifying non-functional overreaching in a previous report [25]. More recently, coronary artery disease patients displayed greater daily fluctuations in self-recorded, home-based heart rate measures preceding a cardiovascular event relative to event-free patients who maintained a stable trend [28]. Though further research is needed to support the current observation of greater LnRMSSD fluctuations Post-SRC, it seems that detection of autonomic dysregulation Post-SRC may require near-daily assessment and that the magnitude of daily fluctuations (e.g., LnRMSSDcv) should be considered [15,18,25,28].

Autonomic dysregulation may be a readily measurable manifestation of the neurometabolic cascade that follows SRC [29,30], but persisting dysfunction is probably mediated by diffuse axonal injury (DAI) that disrupts the structural connectivity within and between brain networks that regulate autonomic processes, emotional responses, and executive functions [12,31,32,33]. The brain’s executive control network (ECN) and central autonomic network (CAN) are closely linked through bidirectional neural signals conveyed through common structural elements, including the anterior cingulate cortex [33,34]. Important internal or external stimuli are identified by the salience network (SN), which deactivates the internally-focused default mode network (DMN) and activates the ECN for a potential need to respond. This process releases the CAN “vagal brake” to prepare for the increased metabolic requirements of ECN engagement and responses to impending demands [34,35]. Dysfunctional network interactions can result in sympathetic hyperactivity in a resting state or during physical exertion. White matter tracts that often exhibit DAI include the genu of the corpus callosum, the anterior corona radiata, and the uncinate fasciculus [36,37]. Microstructural disruptions in these connections among components of the SN, ECN, CAN, and rostral limbic system provide a plausible explanation for loss of normal regulatory control of attention, autonomic processes, and emotional responses [13]. Future research is needed to establish the extent to which HRV reflects neurological status following SRC and whether persistent decrements have relevance for clinical treatment or return to play considerations.

This report is the first to compare serial pre-injury and post-injury HRV measures in a college football player acquired with a method that is feasible for tracking the status of an entire roster of team-members [1,2]. Practitioners responsible for monitoring athletes should be aware that LnRMSSD responses to SRC appear similar to trends associated with stress and training fatigue, which may cause misinterpretation of an athlete’s health and recovery status. Therefore, practitioners should maintain regular communication with the sports medicine staff when an athlete demonstrates alterations in their LnRMSSD trend, which could be due to an unreported SRC. Likewise, sports medicine staff should inform strength and conditioning coaches of head injuries experienced by players so that cardiac-autonomic function can be monitored and interpreted in the appropriate context. A potentially important limitation of our work is lack of HRV measurement in a state of low-level physical exertion, which may reveal persisting abnormalities that are not evident at rest [10,12,13,38]. Another limitation concerns the lack of a control subject. However, comparison of Post-SRC LnRMSSD to a longitudinal baseline may be superior to between-subject comparisons due to the individual nature of LnRMSSD trends, even among teammates of the same position [1]. For example, the LnRMSSDcv presented by the current athlete Post-SRC (5.8%) would still be lower than most other players of the same position group [1] despite elevation from his longitudinal baseline (~3%). Finally, while efforts were made to control for location, environment, time of day, proximity to food or fluid intake, and physical activity, HRV is sensitive to a variety of stimuli [5] that may have contributed to daily variation in the measures.

## 5. Conclusions

This case report demonstrated sustained alterations in the daily HRV pattern of a college football player beyond return to play clearance from a SRC. The HRV responses to SRC appeared similar to trends associated with stress and training fatigue. Because accumulating evidence indicates that SRC can have persisting adverse effects, serial HRV measurements may provide a clinically feasible means to objectively track recovery. In addition to fatigue and recovery status information, the convenient and time-efficient HRV monitoring procedures used in this case report may also be sensitive to physiological responses to SRC that may be relevant for clinical treatment and return to play considerations.

## Figures and Tables

**Table 1:** Change in indicators of status before and after concussion.

Observation Date	Symptom Number ^1^	Symptom Severity ^2^	SAC Score ^3^
Baseline	0	0	27
Day of Injury	8	22	27
Post-Injury Day 1	6	8	-
Post-Injury Day 2	18	36	-
Post-Injury Day 3	4	7	29
Post-Injury Day 4	-	-	-
Post-Injury Day 5	2	2	-
Post-Injury Day 6	0	0	29

^1^ Sport Concussion Assessment Tool (maximum of 22); ^2^ Sport Concussion Assessment Tool (maximum of 132); ^3^ Standardized Assessment of Concussion (perfect score = 30).
